# Boosted Visible-Light Photodegradation of Methylene Blue by V and Co Co-Doped TiO_2_

**DOI:** 10.3390/ma11101946

**Published:** 2018-10-11

**Authors:** Tianping Lv, Jianhong Zhao, Mingpeng Chen, Kaiyuan Shen, Dongming Zhang, Jin Zhang, Genlin Zhang, Qingju Liu

**Affiliations:** School of Materials Science and Engineering, Yunnan Key Laboratory for Micro/nano Materials & Technology, Yunnan University, Kunming 650091, China; Lv_tping@163.com (T.L.); aries88323@163.com (J.Z.); chen_mingpeng@126.com (M.C.); 15198941544@163.com (K.S.); 18341252484@139.com (D.Z.); zhj@ynu.edu.cn (J.Z.)

**Keywords:** V and Co co-doping, visible light response, photocatalytic performance, first-principles calculations

## Abstract

In this work, TiO_2_ photocatalysts, co-doped with transition metal ions vanadium (V) and cobalt (Co) ((V,Co)–TiO_2_), were synthesized by the sol–gel method. The synthesized photocatalysts were characterized by X-ray diffraction (XRD), transmission electron microscopy (TEM), X-ray photoelectron spectroscopy (XPS), nitrogen adsorption and desorption measurement, UV-Vis absorption and photoluminescence spectrum (PL) spectra. The results show that V and Co co-doping has significant effects on sample average crystalline grain size, absorption spectrum, recombination efficiency of photo-induced electron-hole pairs (EHPs), and photocatalytic degradation efficiency of methylene blue (MB). (V,Co)–TiO_2_ photocatalyst exhibits an obvious red shift of the absorption edge to 475 nm. Photocatalytic degradation rate of (V,Co)–TiO_2_ sample for MB in 60 min is 92.12% under a Xe lamp with a cut-off filter (λ > 400 nm), which is significantly higher than 56.55% of P25 under the same conditions. The first principles calculation results show that V and Co ions doping introduces several impurity energy levels, which can modulate the location of the valence band and conduction band. An obvious lattice distortion is produced in the meantime, resulting in the decrease in photo-generated EHP recombination. Thus, (V,Co)–TiO_2_ photocatalyst performance is significantly improved.

## 1. Introduction

The environment pollution, especially water contaminant, has become a serious problem to be urgently overcome. TiO_2_ semiconductor photocatalysis is a kind of pollution treatment technology with great potential, and has been extensively investigated because TiO_2_ photocatalyst is of high photocatalytic activity, inexpensive, and non-toxic and can convert organic pollutants into inorganic substances consuming only solar energy [1,2,3]. However, owing to the relative wide band gap (~3.2 eV), TiO_2_ can just be excited under ultraviolet light with a wavelength of less than 387 nm, which restricts its application [4]. At the same time, the easy recombination of photo-generated EHPs always results in a low photo-quantum yield value of TiO_2_.

To solve this key problem, researchers have utilized a variety of effective methods to modify TiO_2_, including dye-sensitizing [5], noble metal loading [6,7], semiconductor mixing [8,9,10], and ion doping [11,12,13]. Among them, ion doping has been extensively reported as a promising modification method. It is generally believed that ion doping can induce lattice defects [14], make the absorption edges exhibit a red shift [15], and reduce the recombination rate of photo-generated EPHs [16], which further improve the photocatalytic efficiency of material. The dopants mainly include transition metals [17], rare earth metals [18], non-metals [19], and so on. The V-doped TiO_2_ catalytic synthesized by Lin et al. [20] requires 8 h for visible light degradation of MB to reach 90%. Rahimi et al. [21] synthesized Co–TiO_2_ by a sol–gel method and the degradation efficiency of MB was 70% at 3 h under a 300 W tungsten lamp. Chen et al. [22] reported that cobalt- and vanadium-codoped TiO_2_ thin films degradation MB by 75% under UV-light for 24 h. The degradation efficiency of our synthesized (V,Co)–TiO_2_ sample for MB in 60 min was 92.12% under a Xe lamp (λ > 400 nm) with a cut-off filter.

Transition metal ions usually occupy the lattice Ti site and incorporate into the lattice [22]. Amirsalehi and Askari [23] reported that doping cobalt and vanadium affected the crystal growth. This is because the accumulation of cobalt and vanadium ions or the oxygen vacancies generated at the grain boundaries limits the contact of grains and destroys the conventional conditions. The Co-doped TiO_2_ one-dimensional nanostructures were synthesized by a simple solvothermal method reported by De et al. [24]. XPS clearly shows that Ti^4+^ ions are replaced by Co^2+^ ions, demonstrating that Co is incorporated into TiO_2_ lattice to form oxygen vacancies, which greatly changes its optical properties. Through theoretical calculation shows that the impurity level position of V–TiO_2_ is above the valence band maximum (VBM) as well as Co–TiO_2_ is below the conduction band minimum (CBM), which is conducive to the absorption spectrum (S_abs_) red shift. Here, a (V,Co)–TiO_2_ sample was prepared and further studied for its properties. (V,Co)–TiO_2_ photocatalysts with boosted visible-light response were synthesized and further studied for their properties. Simultaneously, the theoretical calculation results were analyzed, and the trend of the changes was found to be in good agreement with the experimental results.

## 2. Materials and Methods

### 2.1. Materials

The tetrabutyl titanate (Ti(OC_4_H_9_)_4_), nitric acid (HNO_3_), ammonium vanadate (NH_4_VO_3_), cobalt nitrate (Co(NO_3_)_2_), acetylacetone, acetone, and absolute ethanol were purchased from Sinopharm Chemical Reagent Co., Ltd. (Shanghai, China). P25 photocatalyst were purchased from Evonik Degussa, Essen, Germany. All reagents were of analytical grade and used without further purification. Deionized (DI) water and hydrolytic inhibitor (a mixture of acetylacetone, acetone, and absolute ethanol) were prepared by our group.

### 2.2. Preparation of Photocatalysts

The (V,Co)–TiO_2_ photocatalyst was synthesized through the sol–gel process as follows: A certain amount of tetrabutyl titanate and hydrolytic inhibitor was slowly added into 75 mL of deionized water under vigorous stirring at 60 °C, and the white suspension was adjusted by 2.5 M HNO_3_ until pH 1.5, labeled as Solution A. A certain amount of NH_4_VO_3_ and Co(NO_3_)_2_ was dissolved wholly in 5 mL of deionized water and labeled as Solution B. Solution B was added to Solution A dropwise, and the fresh solution was strongly stirred for 5–10 h to from collosol. Finally, after being aged, dried, annealed (the calcination process was carried out based on the following processes: heating from room temperature to 500 °C, following by calcining for 2 h before furnace cooling), and ground, the powder of (V,Co)–TiO_2_ photocatalyst was obtained.

### 2.3. Characterization of Photocatalysts

X-ray diffraction (XRD) was characterized on a D/max-2300 diffractometer (Rigaku, Tokyo, Japan) with Cu Kα_1_ radiation (λ = 1.54056 Å) operating at 35 kV and at angles range from 10° to 90° (2θ). Morphology analysis was identified by transmission electron microscopy (TEM) on JEM-2100 microscope (JEOL, Akishima, Japan, 200 kV). X-ray photoelectron spectroscopy (XPS) measurements were performed on a K-Alpha+ spectrometer (Thermo Fisher Scientific, Waltham, MA, USA, 1486.6 eV) with an Al Kα excitation. N_2_ adsorption and desorption measurements were measured on a Quadrasorb-evo equipment (Quantachrome, Boynton Beach, FL, USA) to evaluate the texture of synthesized nanoparticles through the sorption system, and the sample degassed temperature is 300 °C. The S_abs_ response characteristics of the nanoparticles were measured on a UV-Vis spectrophotometer (UV2550, Shimadzu, Kyoto, Japan). In order to research the recombination of photo-generated EHPs of the synthesized nanoparticles, photoluminescence spectrum (PL) was obtained by an FL4500 fluorescence spectrophotometer (Shimadzu, Kyoto, Japan).

### 2.4. Photocatalytic Performance of Photocatalysts

The methylene blue (MB) dye is chosen to evaluate the photocatalytic property of the as-synthesized nanoparticles. A total of 0.2 g of photocatalyst was dispersed in 50 mL of a 0.01 g/L MB solution and mixed homogeneously by stirring. The light irradiation is from a Xe lamp (λ > 400 nm) with a cut-off filter. Before irradiation, the solution was stirred in the dark for 30 min to completely achieve adsorption–desorption equilibrium, and the suspension was then taken out. During irradiation, 4 mL of the suspension was transferred to a centrifuge at an interval of 10 min and centrifuged at 3500 r/min for 30 min. The absorbance value of the MB solution was then measured by a 722 N spectrophotometer at 664 nm and the degradation rate (η) of the MB solution is described by the following equation:

(1) $$\eta =\left[\frac{A_{0}-A_{t}}{A_{0}}\right]\times 100\%$$

where *A*_0_ is the absorbance value of solution after 30 min of dark reaction, and *A_t_* is the absorbance value of solution after *t* min of irradiation.

## 3. Results and Discussion

The orthogonal experiment of photocatalytic degradation rate of MB is presented in Figure S1. According to the orthogonal experimental results, V-doped TiO_2_ with a molar ratio of n(V):n(Ti) = 0.3%, Co-doped TiO_2_ with a molar ratio of n(Co):n(Ti) = 0.1%, and V and Co co-doped TiO_2_ with a molar ratio of n(V):n(Ti) = 0.3% and n(Co):n(Ti) = 0.1% prepared at pH 1.5 and sintered at 500 °C show the best photocatalytic activity, the percentage of degradation is 87.91%, 78.75%, and 92.12%, respectively. Therefore, the V–TiO_2_, Co–TiO_2_, (V,Co)–TiO_2_ photocatalysts discussed below are prepared at above conditions.

### 3.1. Morphology and Structure

**Figure 1:**
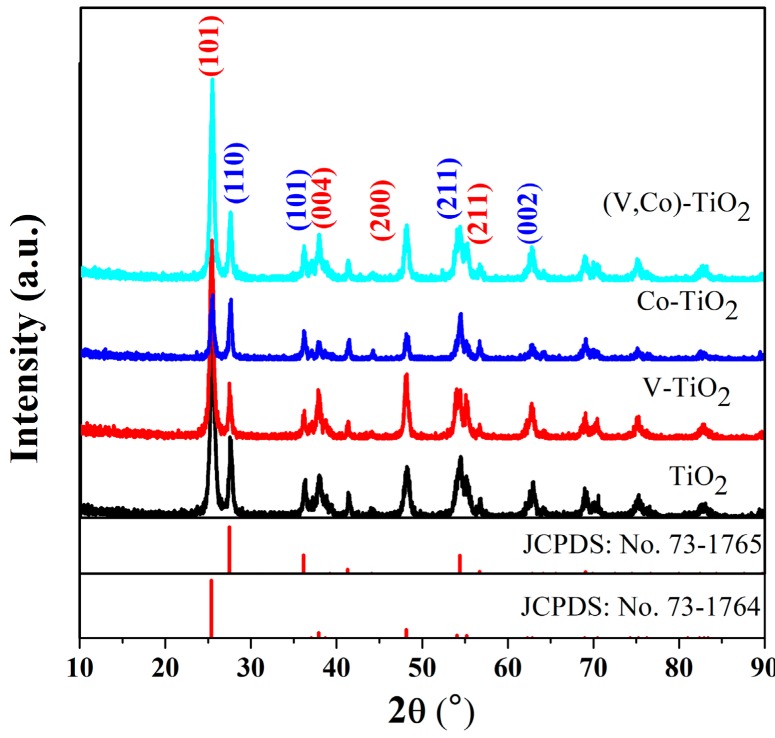
XRD patterns of TiO_2_, V–TiO_2_, Co–TiO_2_, and (V,Co)–TiO_2_ samples.

Figure 1 depicts the XRD patterns of pure TiO_2_, V–TiO_2_, Co–TiO_2_, and (V,Co)–TiO_2_ nanoparticles heat-treated at 500 °C. It is obvious that the XRD pattern of pure TiO_2_ can be assigned to the anatase TiO_2_ (JCPDS No. 73-1764) and rutile TiO_2_ (JCPDS No. 73-1765). Pure TiO_2_ exhibits several diffraction peaks at 25.4°, 37.9°, 48.18°, and 55.24°, corresponding to (101), (004), (200), and (211) planes of anatase phase, and 27.48°, 36.12°, 54.38°, and 62.96°, corresponding to (110), (101), (211), and (002) crystal planes of the rutile phase. In addition, all diffraction peaks of the V–TiO_2_, Co–TiO_2_, and (V,Co)–TiO_2_ samples can be matched with the anatase and rutile phases of pure TiO_2_, but the peak intensity substantially changes. The diffraction pattern shows a sharp peak indicating excellent crystallinity. As shown in Figure 1, the (V,Co)–TiO_2_ sample has the strongest XRD diffraction peak, demonstrating that it has better crystallinity than other samples.

Table 1 is the phase content in the sample calculated according to the Quantitative formula:

(2) $$X_{A}=1/\left(1+1.26I_{R}/I_{A}\right)$$

where *I*_A_ and *I*_R_ are the intensity of XRD peaks of the anatase (101) and rutile (110) planes, respectively [25]. It can be seen from Table 1 that the content of the rutile phase in the Co–TiO_2_ sample increases compared to the pure TiO_2_ prepared under the same conditions, while the rutile phase content of the V–TiO_2_ sample decreases. This indicates that V doping can inhibit the transformation of TiO_2_ from the anatase phase to the rutile phase. However, when V and Co are co-doped, the rutile phase content is less than that of pure TiO_2_ and Co–TiO_2_ photocatalyst, and more than that of V–TiO_2_ photocatalyst.

**Figure 2:**
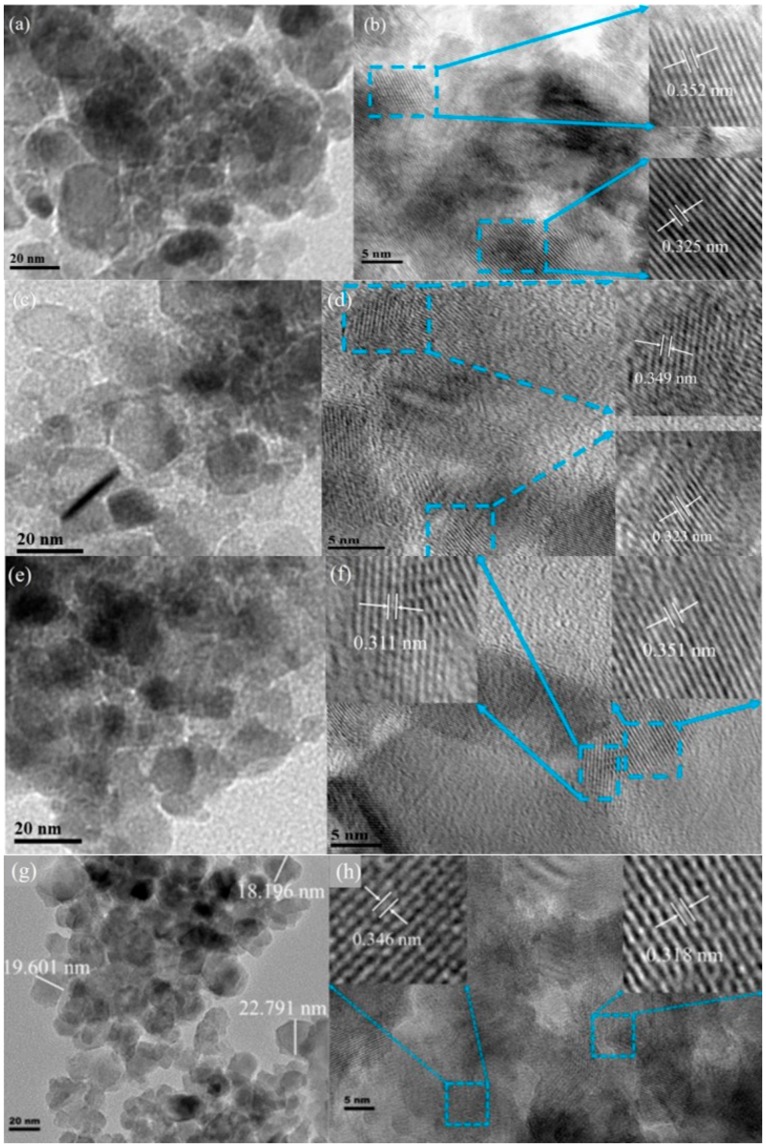
TEM (**a**,**c**,**e**,**g**) and HRTEM (**b**,**d**,**f**,**h**) images of the TiO_2_, V–TiO_2_, Co–TiO_2_, and (V,Co)–TiO_2_ samples.

The morphology of TiO_2_, V–TiO_2_, Co–TiO_2_, and (V,Co)–TiO_2_ samples are shown in Figure 2. The TEM shows that the samples consist of sphere-like homogeneous particles with the average diameter of 20 nm, but they significantly agglomerated. Clear lattice fringes can be seen from high-resolution transmission electron microscopy (HRTEM) images of TiO_2_ (Figure 2b), V–TiO_2_ (Figure 2d), Co–TiO_2_ (Figure 2f), and (V,Co)–TiO_2_ (Figure 2h) samples, showing outstanding crystallinity, which are composed of rutile and anatase phases. Figure 2b demonstrates the lattice planes of anatase (101) with d-spacing of 0.352 nm and rutile (110) with d-spacing of 0.325 nm of pure TiO_2_. Compared to pure TiO_2_, the lattice spacing of the doped samples changes, which indicates that V and Co ions have been doped into TiO_2_ crystal lattice and can cause lattice distortion.

**Figure 3:**
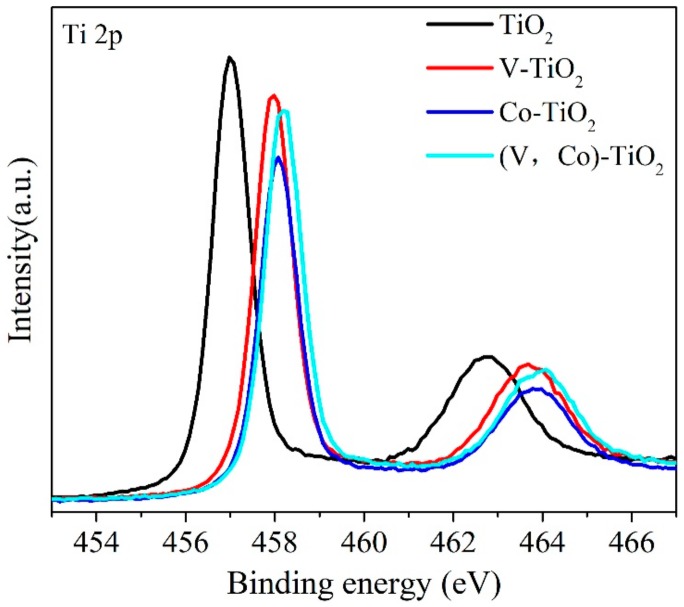
Ti 2p XPS spectra of TiO_2_, V–TiO_2_, Co–TiO_2_, and (V,Co)–TiO_2_ samples.

The TiO_2_, V–TiO_2_, Co–TiO_2_, and (V,Co)–TiO_2_ samples are further measured by XPS. Figure 3 exhibits the XPS spectra of Ti 2p characteristic peaks. For pure TiO_2_, two characteristic peaks located at 457 and 462.78 eV could be assigned to Ti 2p_3/2_ and Ti 2p_1/2_, respectively. The position of the Ti 2p characteristic peak of V–TiO_2_ and Co–TiO_2_ is obviously changed. In particular, the Ti 2p_3/2_ and Ti 2p_1/2_ peak of (V,Co)–TiO_2_ sample are shifted towards higher binding energy to 458.18 and 464.08 eV, respectively. Because the Pauling electronegativity of Ti^4+^ (1.54) is smaller than that of V^4+^ (1.63) and Co^2+^ (1.88), the electron of Ti^4+^ will transfer to V^4+^ and Co^2+^, resulting in the electron number decreasing in Ti^4+^, and subsequently increases the binding energy [26]. At the same time, the Ti 2p binding energies of (V,Co)–TiO_2_ increase, indicating that some Ti atoms are substituted by V and Co ions in the lattice, which may produce more V–O–Ti, Co–O–Ti, and V–O–Co structures [27].

**Figure 4:**
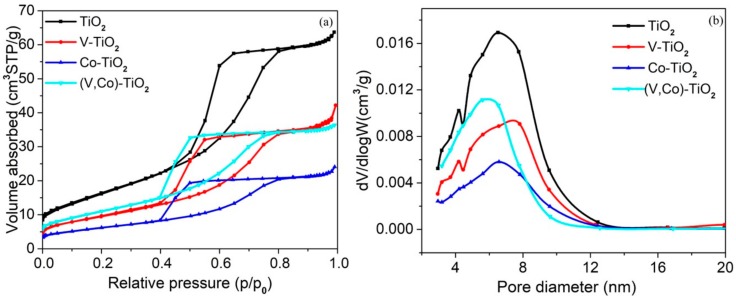
Nitrogen adsorption–desorption isotherm (**a**) and the corresponding pore size distribution (**b**) for TiO_2_, V–TiO_2_, Co–TiO_2_, and (V,Co)–TiO_2_ samples.

The nitrogen adsorption–desorption isotherm was performed to determine the specific surface area and porous structure of TiO_2_, V–TiO_2_, Co–TiO_2_, and (V,Co)–TiO_2_ samples, and the relevant results are shown in Figure 4. We can observe that the isotherm of four samples exhibits a type IV shape with an H3 hysteresis loop in the region of 0.4–0.8 p*/*p_0_, according to the IUPAC classification [28]. This indicates that the as-synthesized sample is a mesoporous material, and this desorption hysteresis phenomenon is related to the shape and size of the pore. It can be seen from the pore size distribution diagram of Figure 4b that pure TiO_2_ photocatalyst has a wide pore size distribution, which is disadvantageous for the adsorption property of MB. The (V,Co)–TiO_2_ photocatalyst has a narrow pore size distribution, which makes it easier to capture MB and helps to increase adsorption property for MB and photocatalytic reaction, thereby improving its photocatalytic degradation efficiency [29]. As can be seen from Table 2, the samples differ in physical properties. (V,Co)–TiO_2_ sample has the smallest pore size, but its specific surface area (S_BET_) and pore volume are smaller than that of pure TiO_2_. This indicates that the performance of (V,Co)–TiO_2_ photocatalyst does not entirely depend on specific surface area, pore volume, and pore size.

### 3.2. Optical Property

**Figure 5:**
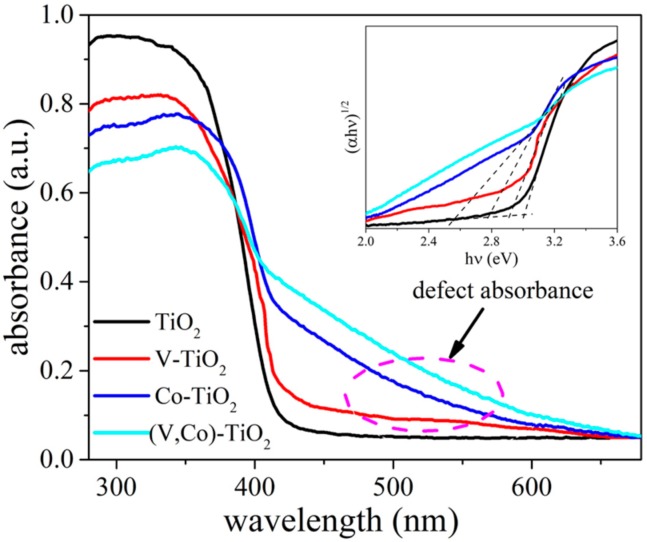
UV-Vis spectra of TiO_2_, V–TiO_2_, Co–TiO_2_, and (V,Co)–TiO_2_ samples, inset displays the corresponding Tauc plots, as well as the band gap values.

Figure 5 presents the optical S_abs_ of TiO_2_, V–TiO_2_, Co–TiO_2_, and (V,Co)–TiO_2_ samples in the range of 300–700 nm. Compared with TiO_2_, all the S_abs_ of the V–TiO_2_, Co–TiO_2_, and (V,Co)–TiO_2_ samples show a red shift, and the red shift is more obvious for the (V,Co)–TiO_2_ sample, moving from 405 to 475 nm. The energy required to make electrons transition from a valence band to a conduction band decreases, and the absorption of visible light increases [30]. From the marked circle of Figure 5, it was detected that the doped TiO_2_ showed a clear band tailing. Because the absorption of electrons from the defect state always leads to light absorption, the intensity of the band tailing can directly reflect the number of defect states [31]. It can be seen that (V,Co)–TiO_2_ has more defect states. Tauc plots can determine the band gap energy of TiO_2_ and doped TiO_2_ samples. The band gap value of TiO_2_ is 3.01 eV, with the doping of V and Co ions, the band gap decreased to 2.91 eV (V–TiO_2_), 2.77 (Co–TiO_2_), and 2.56 eV ((V,Co)–TiO_2_), which is in agreement with the trend of theoretically calculated band gap values.

**Figure 6:**
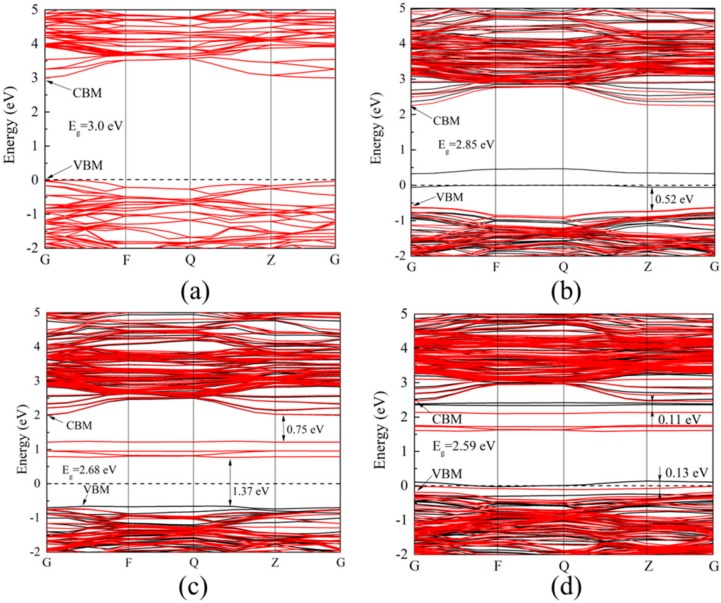
The band structures of (**a**) TiO_2_, (**b**) V–TiO_2_, (**c**) Co–TiO_2_, and (**d**) (V,Co)–TiO_2_.

**Figure 7:**
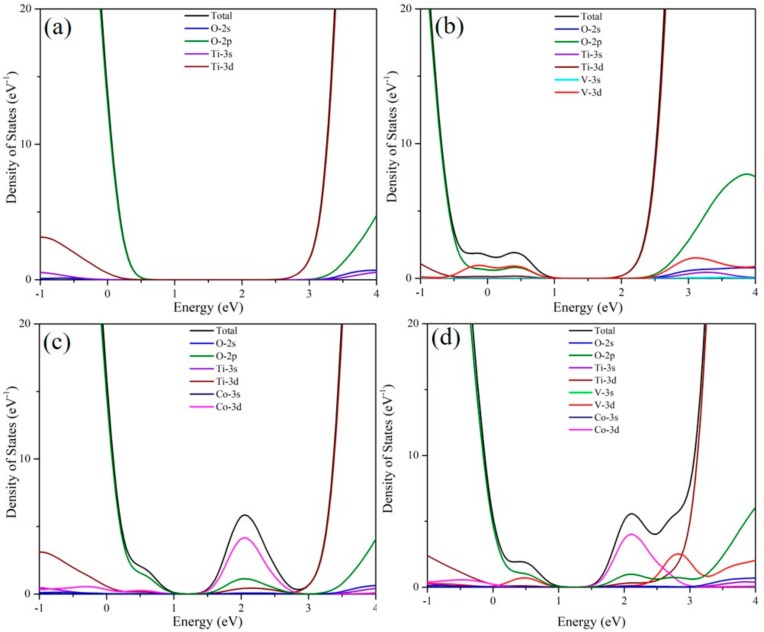
The DOS of (**a**) TiO_2_, (**b**) V–TiO_2_, (**c**) Co–TiO_2_, and (**d**) (V,Co)–TiO_2_.

To investigate the effect of doping V and Co ions on the electronic structure of anatase TiO_2_, a set of density functional theory (DFT) calculations were carried out. Figure 6 and Figure 7 represent the energy band structure and density of states (DOS) of four TiO_2_ photocatalytic systems, respectively. The calculations in this paper were completed by cambridge sequential total energy package (CASTEP) module in Materials Studio software (version: MS6.0), using the generalized gradient approximation (GGA) method under the framework of density functional theory. The plane wave cut-off energy set was 400 eV. For obtaining an accurate electronic structure, the GGA + U (U = 5 eV) method is used to overcome the disadvantages of GGA [32]. In the Brillouin zone, the k-points grid sampling was set to 2 × 2 × 2. The convergence parameter settings were as follows: 5.0 × 10^−5^ eV/atom for maximum energy tolerance, 0.10 eV/Å for maximum force tolerance, 0.20 GPa for maximum stress tolerance, and 5.0 × 10^−3^ Å for maximum displacement tolerance. Figure 6a illustrates that the band gap of pure TiO_2_ is calculated to be 3.00 eV, which is very close to the experimental result. The band gaps of doping TiO_2_ are smaller than that of pure TiO_2_, in terms of V–TiO_2_ (2.85 eV), Co–TiO_2_ (2.68 eV), and (V,Co)–TiO_2_ (2.59 eV). The impurity levels of V–TiO_2_ and Co–TiO_2_ locate between the forbidden bands, and the band gaps are reduced after doping, leading to an S_abs_ red shift. Meanwhile, multiple impurity levels are induced in the forbidden band, which can facilitate the absorption of visible light by the TiO_2_ photocatalyst [33]. However, the excessive doping concentration will produce more recombination center for the EHPs. Compared with V–TiO_2_ and Co–TiO_2_, the impurity levels in (V,Co)–TiO_2_ are closer to CBM and VBM. Notably, the band gap of (V,Co)–TiO_2_ is significantly smaller than that of V–TiO_2_ and Co–TiO_2_, and the dipole moment of (V,Co)–TiO_2_ (3.412 Debye) is significantly larger than that of V–TiO_2_ (0.041 Debye) and Co–TiO_2_ (0.042 Debye), which not only contributes to the absorption of visible light but also prevents the recombination of the EHPs. In addition, the impurity level above the Fermi level can capture photo-generated electrons, further reducing the EHP recombination and improve the photocatalytic efficiency [34].

**Figure 8:**
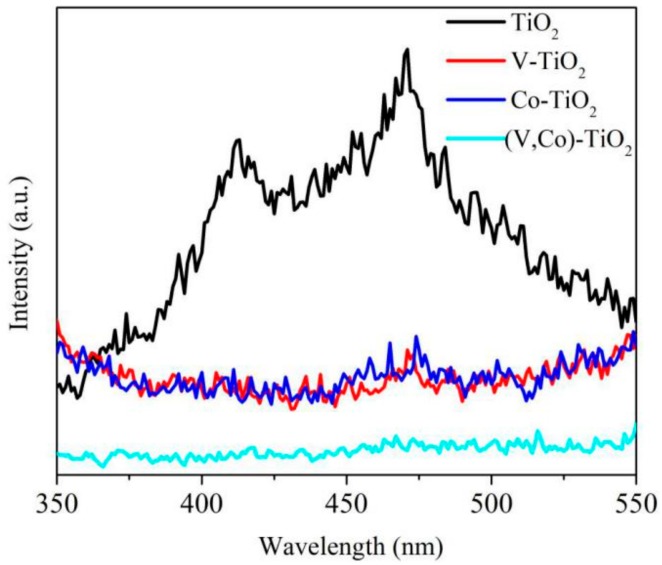
PL spectra of TiO_2_, V–TiO_2_, Co–TiO_2_, and (V,Co)–TiO_2_ samples.

For investigating the recombination of the EHPs, PL spectra of pure TiO_2_, V–TiO_2_, Co–TiO_2_, and (V,Co)–TiO_2_ samples were carried out in Figure 8. Since the PL emission is the result of the recombination of the excited EHPs, the lower the PL intensity of the sample, the lower the recombination rate [35]. Compared to the PL spectra of pure TiO_2_, the fluorescence intensity of (V,Co)–TiO_2_ sample is significantly weaker, indicating that (V,Co)–TiO_2_ can effectively inhibit the recombination of the EHPs. Due to the V and Co ions doping, the lattice of TiO_2_ is distorted, so that the positive and negative charge centers of the titanium octahedron [TiO_6_] do not coincide and the internal dipole moment is generated. The local electric field generated by the internal dipole moment causes the EHPs to be effectively separated [36]. The photoluminescence intensity of (V,Co)–TiO_2_ nanoparticles is weaker than that of V–TiO_2_ and Co–TiO_2_ sample. As a result, the lattice distortion of TiO_2_ is changed after co-doping of V and Co ions, resulting in a larger internal dipole moment and a further decrease in the recombination rate of photo-generated carriers, thereby facilitating the separation process of charges.

### 3.3. Photocatalytic Property

**Figure 9:**
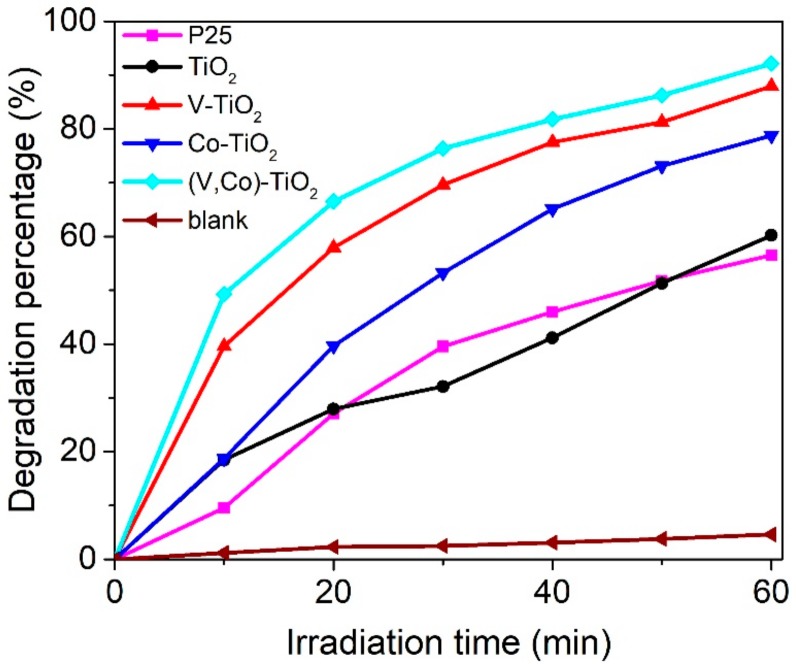
Degradation rate of MB over the P25, TiO_2_, V–TiO_2_, Co–TiO_2_, and (V,Co)–TiO_2_ samples and without the catalyst samples.

Photocatalytic degradation rate of MB is presented in Figure 9. It is observed that pure TiO_2_, V–TiO_2_, and Co–TiO_2_ samples exhibit a better photocatalytic efficiency than that of P25. The rate of the (V,Co)–TiO_2_ photocatalyst for photocatalytic degradation of MB for 60 min under a Xe lamp (λ > 400 nm) with a cut-off filter is 92.12%, which is significantly better than 56.55% of the P25 photocatalyst, the V–TiO_2_ and Co–TiO_2_ samples. The Mn/TiO_2_-WACF photocatalytic composite material synthesized by Ma et al. [37] requires 200 min for visible light degradation of MB to reach 90%. Shang et al. [38] prepared ZnO–Ag–TiO_2_ NTAs photocatalytic materials by the one-step anodization method. Its photodegradation efficiency for MB in 120 min was 86%. The rate of (V,Co)–TiO_2_ photocatalyst for photocatalytic degradation of MB for 60 min under visible-light is 92.12%, which is significantly better than that of the Mn/TiO_2_–WACF and ZnO–Ag–TiO_2_ NTAs photocatalysts. According to the previous analysis, the photocatalytic degradation rate of the (V,Co)–TiO_2_ is improved for the following two reasons. First, the S_abs_ of (V,Co)–TiO_2_ is red-shifted, and the absorption of visible light is enhanced. Secondly, the lattice distortion that occurs after the co-doping of V and Co ions produce a stronger internal dipole moment, which effectively separates the EHPs, so the photocatalytic degradation property of the (V,Co)–TiO_2_ is better than that of the other two samples.

**Figure 10:**
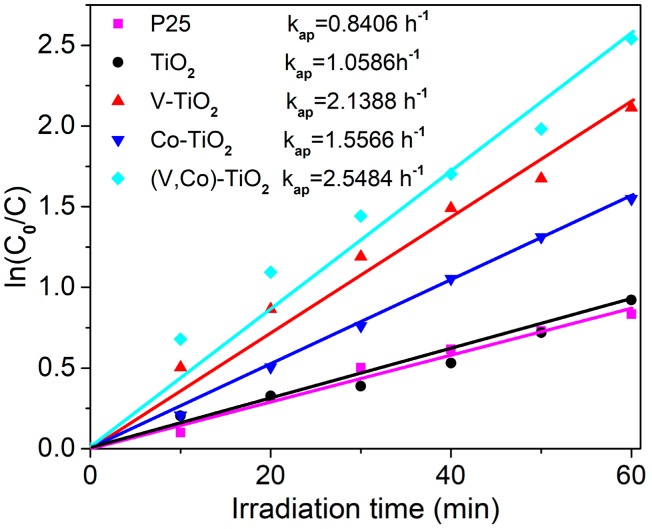
The kinetics of MB photocatalytic degradation over the P25, TiO_2_, V–TiO_2_, Co–TiO_2_, and (V,Co)–TiO_2_ samples.

It is known that photocatalytic reaction follows the classical Langmuir–Hinshelwood kinetic model. As shown in Figure 10, the kinetics of the MB photodegradation by P25, TiO_2_, V–TiO_2_, Co–TiO_2_, and (V,Co)–TiO_2_ photocatalysts are fitted by the pseudo-first-order kinetic equation [39]:

(3) $$\ln\left(\frac{C_{0}}{C}\right)=k_{\mathrm{ap}}t$$

where *C*_0_ is the initial concentration value, and *C* is the concentration of MB after light irradiating at *t* min, *k*_ap_ is the first-order apparent efficiency constant, determined by the slope of the line, and indicates the photocatalytic activity of the photocatalyst. Obviously, for these three photocatalysts, the rate constants of TiO_2_, V–TiO_2_, Co–TiO_2_, and (V,Co)–TiO_2_ are 1.0586 h^−1^, 2.1388 h^−1^, 1.5566 h^−1^, and 2.5484 h^−1^, respectively, which are 1.2, 2.5, 1.8, and 3.0 times higher than that of P25 (0.8406 h^−1^). It can be seen that the (V,Co)–TiO_2_ photocatalyst has the maximum rate constant for photodegradation of MB, and the highest degradation efficiency in the reactions.

In summary, we have rationally designed and implemented the preparation of (V,Co)–TiO_2_ photocatalyst and achieved efficient visible-light response. This is due to the fact that vanadium and cobalt ions replace the lattice sites of titanium, resulting in lattice distortion of TiO_2_. The doping of vanadium and cobalt ions can narrow the band gap of (V,Co)–TiO_2_ photocatalyst, reduce the recombination rate of photo-generated electron-hole pairs, and red-shift the absorption band edge, thereby enhancing its absorption of visible-light and improving its photocatalytic performance in the visible region.

## 4. Conclusions

(V,Co)–TiO_2_ photocatalyst was successfully synthesized through a simple sol–gel method, proper doping amount of V and Co can reduce recombination probability of photo-generated EHPs, and makes absorption band edge red shift, enhanced absorption of visible light and improved photocatalytic activity. Compared with TiO_2_ (405 nm), all the S_abs_ of the V–TiO_2_ (426 nm), Co–TiO_2_ (447 nm) and (V,Co)–TiO_2_ (475 nm) samples show red-shift. The red-shift of absorption band edge of (V,Co)–TiO_2_ is the largest, of which the band gap is the narrowest, photo-generated electron-hole pairs have lowest recombination rate, and extend the lifetime of photo-generated carriers, thus increase photocatalytic efficiency. Photocatalytic degradation rate of MB in 60 min is 92.12% under a Xe lamp (λ > 400 nm) with a cut-off filter, which is significantly higher than 56.55% of P25 under the same time conditions. The theoretical calculation results show that the band gap of (V,Co)–TiO_2_ photocatalytic system is significantly reduced, and the impurity level is generated between the band gaps, which is beneficial to red shift of the absorption band edge, and thus greatly improve the photocatalytic performance.

## Figures and Tables

**Table 1:** Anatase and rutile content of TiO_2_, V–TiO_2_, Co–TiO_2_, and (V,Co)–TiO_2_ samples.

Samples with Different Doping Element	TiO_2_	V–TiO_2_	Co–TiO_2_	(V,Co)–TiO_2_
Anatase content (%)	64.07	78.62	47.07	70.05
Rutile content (%)	35.93	21.38	52.93	29.95

**Table 2:** S_BET_, pore volume, and average pore size of TiO_2_, V–TiO_2_, Co–TiO_2_, and (V,Co)–TiO_2_ samples.

Sample	SBET (m^2^ g^−1^)	Pore Volume (cm^3^ g^−1^)	Average Pore Diameter (nm)
V–TiO_2_	35.270	0.056	7.4
Co–TiO_2_	22.812	0.033	6.5
(V,Co)–TiO_2_	40.865	0.051	5.5
TiO_2_	61.314	0.093	6.4

## Supplementary Materials

The following are available online at http://www.mdpi.com/1996-1944/11/10/1946/s1, Figure S1: degradation percentage of MB by V and Co doped TiO_2_ samples prepared at pH 1.5 and sintered at 500 °C. (a) V–TiO_2_ of different doping quantities, (b) Co–TiO_2_ of different doping quantities, (c) (V, Co)–TiO_2_ of different doping quantities, Figure S2: five recyclable visible-light catalytic degradation experiments using (V, Co)–TiO_2_ photocatalyst in 0.01g L^−1^ MB solution.
