# Supplementary material for: Boosted Visible-Light Photodegradation of Methylene Blue by V and Co Co-Doped TiO2
Source: Materials (Basel). 2018 Oct 11;11(10):1946. doi: 10.3390/ma11101946 (PMC6213182; doi:10.3390/ma11101946)
Supplement: Supplementary file 1 [file materials-11-01946-s001.pdf]

Supplementary Information

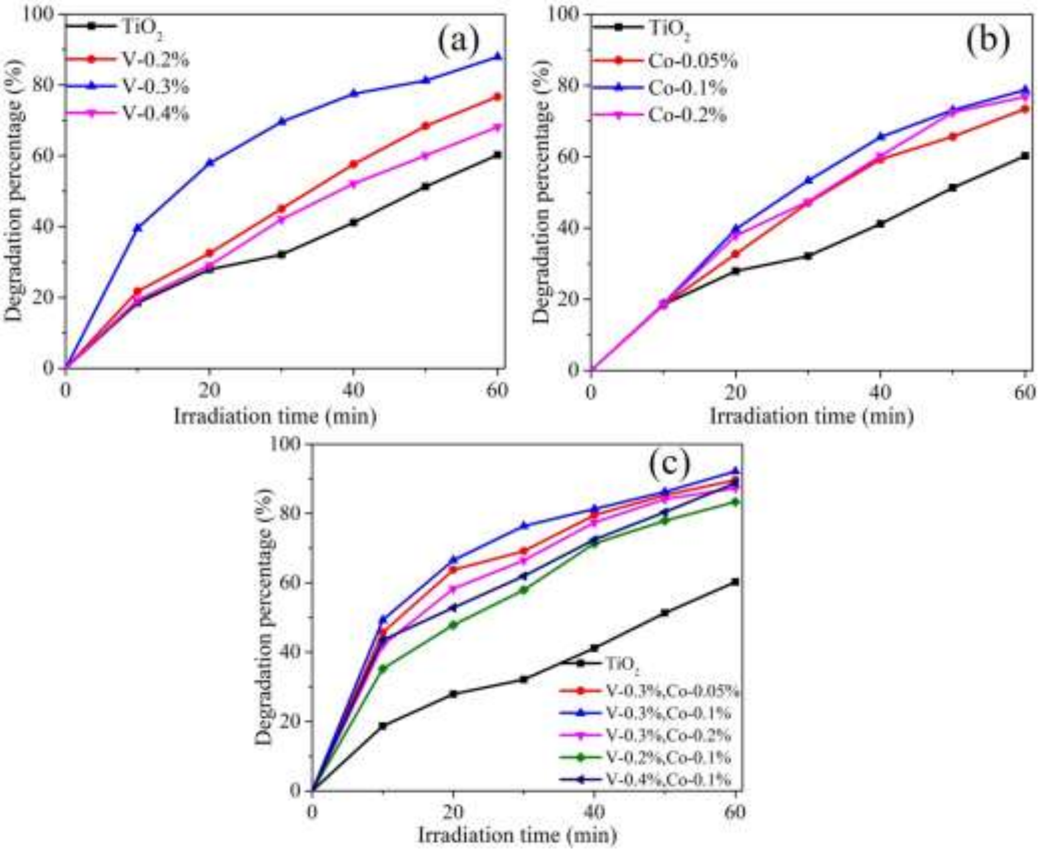

Figure S1. Degradation percentage of MB by V and Co doped TiO_2 samples prepared at pH 1.5 and sintered at 500 °C. (**a**) V–TiO_2 of different doping quantities; (**b**) Co–TiO_2 of different doping quantities; (**c**) (V, Co)–TiO_2 of different doping quantities.

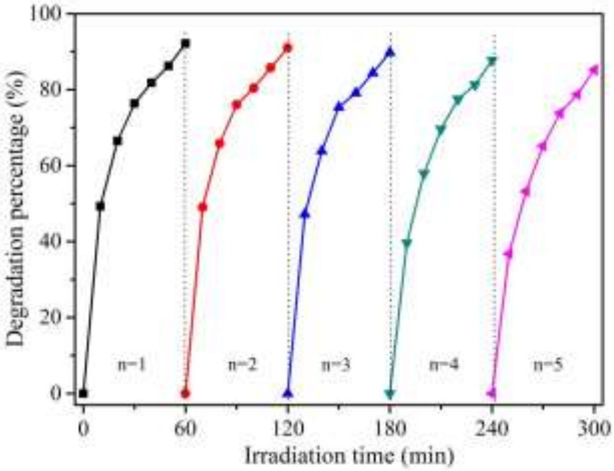

Figure.S2. Five recyclable visible-light catalytic degradation experiments using (V, Co)–TiO_2 photocatalyst in 0.01g L^{-1} MB solution.

© 2018 by the authors. Submitted for possible open access publication under the terms and conditions of the Creative Commons Attribution (CC BY) license (http://creativecommons.org/licenses/by/4.0/).
